# Enhanced Monitoring of Sleep Position in Sleep Apnea Patients: Smartphone Triaxial Accelerometry Compared with Video-Validated Position from Polysomnography

**DOI:** 10.3390/s21113689

**Published:** 2021-05-26

**Authors:** Ignasi Ferrer-Lluis, Yolanda Castillo-Escario, Josep Maria Montserrat, Raimon Jané

**Affiliations:** 1 Institute for Bioengineering of Catalonia (IBEC), Barcelona Institute of Science and Technology (BIST), 08028 Barcelona, Spain; ycastillo@ibecbarcelona.eu (Y.C.-E.); 2 Centro de Investigación Biomédica en Red de Bioingeniería, Biomateriales y Nanomedicina (CIBERBBN), 28029 Madrid, Spain; 3 Department of Automatic Control (ESAII), Universitat Politècnica de Catalunya-Barcelona Tech (UPC), 08028 Barcelona, Spain; 4 Sleep Lab, Pneumology Service, Hospital Clínic de Barcelona, 08036 Barcelona, Spain; jmmontserrat@ub.edu (J.M.M.); 5 Centro de Investigación Biomédica en Red de Enfermedades Respiratorias (CIBERES), 28029 Madrid, Spain

**Keywords:** accelerometry, biomedical signal processing, mHealth, monitoring, sleep apnea, sleep position, smartphone

## Abstract

Poor sleep quality is a risk factor for multiple mental, cardiovascular, and cerebrovascular diseases. Certain sleep positions or excessive position changes can be related to some diseases and poor sleep quality. Nevertheless, sleep position is usually classified into four discrete values: supine, prone, left and right. An increase in sleep position resolution is necessary to better assess sleep position dynamics and to interpret more accurately intermediate sleep positions. This research aims to study the feasibility of smartphones as sleep position monitors by (1) developing algorithms to retrieve the sleep position angle from smartphone accelerometry; (2) monitoring the sleep position angle in patients with obstructive sleep apnea (OSA); (3) comparing the discretized sleep angle versus the four classic sleep positions obtained by the video-validated polysomnography (PSG); and (4) analyzing the presence of positional OSA (pOSA) related to its sleep angle of occurrence. Results from 19 OSA patients reveal that a higher resolution sleep position would help to better diagnose and treat patients with position-dependent diseases such as pOSA. They also show that smartphones are promising mHealth tools for enhanced position monitoring at hospitals and home, as they can provide sleep position with higher resolution than the gold-standard video-validated PSG.

## 1. Introduction

Poor sleep quality or disturbed sleep is associated with multiple health complications, including mental disorders [1,2,3] and cardiovascular and cerebrovascular diseases [4,5,6]. Obstructive sleep apnea (OSA) is one of the most common diseases affecting sleep quality. It is a condition caused by repeated episodes of upper airway collapse and obstruction during sleep, associated with arousals with or without oxygen desaturation [7,8,9,10]. These episodes can be of total occlusion (apnea) or partial occlusion (hypopnea) of the air pathways, leading to a total or partial reduction of the airflow [11].

Sleep position is an important factor to consider when analyzing sleep quality and diagnosing OSA. Previous studies highlight the role of sleep position in the appearance of apneas, a phenomenon known as positional OSA (pOSA) [12,13]. In addition, the effect of sleep position on the ocular surface has also been assessed and related to the occurrence of different diseases such as dry eye, ocular hypertension, or glaucoma [14,15,16].

Multiple different techniques can be used to determine sleep quality. For instance, questionnaires, such as the Pittsburgh sleep quality index (PSQI) [17], the Epworth sleepiness scale [18], the STOP-Bang questionnaire [19] and the Berlin questionnaire [20], among others [21], are commonly used tools for assessing sleep quality. Questionnaires and self-reported information aim to predict the severity of multiple symptoms related to poor sleep quality, including snoring associated with OSA [22,23], daytime sleepiness or fatigue, which predict the risk of accidents [24]. Yet, questionnaires and self-reported information fail to properly determine the different sleep positions [25].

Other techniques, such as polygraphy and polysomnography (PSG), aim to assess the sleep quality in a more objective manner. These techniques determine the apnea-hypopnea index (AHI), which is an indicator related to the severity of OSA. This indicator calculates the number of apneas and hypopneas per hour of sleep. According to the American Academy of Sleep Medicine (AASM) [11], the AHI classifies patients into four different categories: healthy (AHI < 5); mild OSA (5 ≤ AHI < 15); moderate OSA (15 ≤ AHI < 30) and severe OSA (AHI ≥ 30). Home respiratory polygraphy (HRP) uses less sensors than PSG, which is the gold-standard method of assessing sleep quality and position. Since PSG includes video-surveillance, it is used to validate the sleep position. However, it has some limitations: the sleep position is classified into only four categories (supine, prone, left and right); the level of sleep comfort is changed (different bed and pillow, and full of PSG wires); and the prevalence of the supine sleep position in PSG studies [26,27], which can affect the diagnosed severity of OSA. Therefore, many patients remain undiagnosed and untreated [28,29], worsening their long-term consequences.

There exist multiple treatments for sleep apnea [30]. These treatments range from more invasive options, such as surgical correction of the air pathways structures to prevent occlusions, to less invasive treatments, such as continuous positive airway pressure (CPAP) [31] or positional therapy [32], which aims to force the patient to sleep in non-supine sleep positions to reduce the AHI index and increase the sleep quality. Yet, self-reported sleep position is unreliable and objective measures are required [25].

In recent years, smartphones have been suggested as potential candidates with which to monitor sleep apnea, due to their prevalence and the range of embedded sensors [33]. The appearance of sensing technologies related to sleep has also increased in the last 10 years [34]. Multiple approaches using different combinations of these smartphone sensors to monitor sleep apnea have been documented, including previous work by our group. Some studies have made use of the embedded microphone sensor to diagnose and monitor sleep apnea [35,36,37,38], and others have used the embedded accelerometry sensor of the smartphone to monitor sleep apnea and position [39,40,41]. The treatment of pOSA has also been researched with smartphone applications which vibrated when the patient slept in a supine position, to promote lateral sleep positions [42]. Besides smartphones, there have also been multiple attempts to develop applications which could be used to monitor sleep apnea with minimal sensors, which include accelerometry [43,44,45], audio [35,46], pulse oximetry [47,48] and other sensors [49,50,51]. Finally, recent studies have shown the importance of a higher-resolution sleep position for the diagnosis of pOSA [52], since there could exist pOSA variability within the four clinically used sleep position categories (supine, prone, left and right).

The aim of this study is to propose a higher resolution sleep position detector using triaxial accelerometry from smartphones. To accomplish this, we divided our study into three separate tasks: (1) study the feasibility of smartphones as sleep position monitors and develop algorithms to retrieve the sleep position angle from smartphone accelerometry; (2) monitor the sleep position angle in adult patients with obstructive sleep apnea (OSA); and (3) discretize the sleep angle into the four sleep positions to compare it with the sleep position from the video-validated polysomnography (PSG) and analyze the OSA event distribution (pOSA).

## 2. Materials and Methods

### 2.1. Hospital Database and Acquisition Protocol

**Figure 1:**
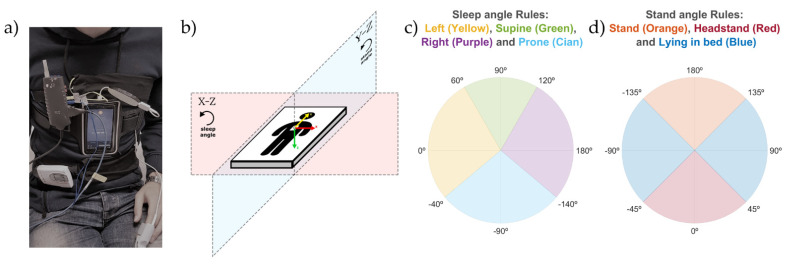
Picture of the smartphone and PSG device placement (**a**) and representation of the smartphone placement (**b**) including the direction of the accelerometry X axis (red), Y axis (yellow) and Z axis (green) as well as the X-Z plane, where the sleep angle is calculated, and the Y-Z plane, where the stand angle is calculated. Explanation of the rules used to discretize the sleep angle (**c**) and stand angle (**d**). The sleep angle was classified into the four sleep positions: supine, prone, left and right. The stand angle into two categories: standing (either stand or headstand) and lying. The stand category also had a second constraint (3).

The acquisition protocol used for all of the experiments in this study was approved by the ethics committee from the Hospital Clínic of Barcelona and conducted with OSA patients. Two different devices were used simultaneously to record the sleep position during night-time acquisitions at the Sleep Lab in the Hospital Clínic of Barcelona. The reference device, a ‘Grael PSG’ (Compumedics, Melbourne, Australia), recorded the sleep position using the company’s proprietary algorithms at a sampling frequency of 32 Hz. The equipment was positioned according to the standard procedure. The second device, a Samsung S5 SM-G900F Android 6.0.1 smartphone (Samsung, Seoul, South Korea), was used as the test device. It recorded triaxial accelerometer data with its embedded MPU-6500 sensor at a sampling frequency of 200 Hz using the Sensors Logger application [53]. This application saves the data in the smartphone memory as a text file. The smartphone was placed over the sternum and held in position with an elastic strap as seen in Figure 1a, based on the configuration proposed by Nakano et al. [35], which has been tested successfully in previous publications of our group [36,37,38,40,41]. This configuration resulted in the smartphone triaxial accelerometry providing positive values when accelerations occurred from right to left (X-axis), from toe to head (Y-axis), and from front to back (Z-axis), as seen in Figure 1b. The smartphone was used in flight mode, and with the Wi-Fi and Bluetooth options disabled.

The acquisition protocol described above was used to register 20 different OSA patients. The inclusion criteria were based upon medical examination of the medical doctors from the sleep lab in the Hospital Clínic of Barcelona. This medical examination aimed to obtain a gender-age-balanced database with patients with different AHI severity indexes. At random, 20 patients were included with mild to severe obstructive sleep apnea diagnosed by a full PSG performed in the previous 2 months. The exclusion criteria were: Patients with hypoventilation, central sleep apnea (Cheyne Stokes), uvulopalatopharyngoplasty, very severe nasal obstruction or in those who refused their consent. For technical reasons, one of the patients had to be discarded. Therefore, the database used for analysis is composed of 9 men and 10 women, with an average age of 60 (38–78) and an average AHI of 34 (6–70). Automatic PSG sleep positions were video-validated by sleep technicians, and automatic and validated positions were both saved separately. The patients were asked to rest in the supine position for some minutes at the beginning of the test. The minimum final valid duration for each patient was five hours of sleep.

### 2.2. Signal Preprocessing

The automatic and video-validated sleep positions obtained from the PSG were exported in .edf file format and the smartphone triaxial accelerometry was stored in .txt files. The three position signals were processed and analyzed using custom developed algorithms in MATLAB r2019b (Mathworks Inc., Natick, MA, USA).

The PSG signals were upsampled to 200 Hz and the signals from the two devices were automatically synchronized with the beginning and end timestamps of each file. Initial and final regions with no overlap between signals were discarded. The first and last 10 min were also removed due to artifacts related to equipment placement and displacement, respectively.

### 2.3. Sleep Position Monitoring: Sleep and Stand Angles

The algorithm described in this subsection aims to retrieve the sleep position angle, which allows us to obtain a more precise sleep position with better control of the position shifts. To calculate the sleep angle from the triaxial accelerometry the following steps were performed:

1. Each of the triaxial accelerometer signals (X, Y, and Z) was filtered with a median filter with a window of 60 s to remove high-frequency noise and keep the signal baseline containing the gravity acceleration.
2. For each sample of the triaxial accelerometry, two different angles were calculated using (1): the sleep position angle and the stand angle.
  (1) $$\mathrm{Angle}\;(º)=\frac{180}{\Pi }\cdot \mathrm{acos}\left(\frac{\vec{a}\cdot \vec{b}}{\left|\vec{a}\right|\cdot \left|\vec{b}\right|}\right)\cdot sign(c)$$
  2.1. The sleep position angle was used to determine the four sleep positions (supine, prone, left and right). It was calculated by (1), where $\vec{a}=\left(X_{n},Z_{n}\right)$, with $X_{n}$ and $Z_{n}$ being the values of the X and Z axis of the triaxial accelerometry at each specific timestamp; $\vec{b}=\left(1,\;0\right)$, which is a static reference aligned with the left sleep position; and $c={\;Z}_{n}$, to be able to differentiate supine and prone positions. This angle explains the orientation of the accelerometry in the X-Z plane as shown in Figure 1b.
  2.2. The stand angle was used to determine whether the patient is in a standing or lying position. It was calculated by (1), where $\vec{a}=\left(Y_{n},Z_{n}\right)$, with $Y_{n}$ and $Z_{n}$ being the values of the *Y* and *Z* axis of the triaxial accelerometry at each specific timestamp; $\vec{b}=\left(1,\;0\right)$, which is a static reference aligned with the stand position; and $c={\;Z}_{n}$, to be able to differentiate stand and headstand positions. This angle explains the orientation of the accelerometry in the Y-Z plane, as shown in Figure 1b.
3. Two different corrections were made to ensure that the angles calculated provided the real patient position:
  3.1. Module correction: to avoid unreal position angle calculations, due to small angle value variations in close-to-zero module vectors, both the sleep and stand angle values were replaced with their last module-correct angle value if the following criteria was found true:
    (2) $$\frac{\left|\vec{a}\right|}{\left|\vec{r}\right|}<Thld1$$
    where Thld1 is a threshold with a value of 0.5; $\vec{a}$ belongs to the $\vec{a}$ vectors declared for the sleep and stand angles in the steps 2.1 and 2.2 of this subsection; and $\vec{r}=\left(X_{n},Y_{n},Z_{n}\right)$, with $X_{n}$, $Y_{n}$ and $Z_{n}$ being the values of the X, Y and Z axis of the triaxial accelerometry at each specific timestamp.
  3.2. Initial position correction: the supine positions in the first 10 min of the triaxial accelerometer signals were used to automatically self-correct the differences in smartphone placement due to anatomical variations in patients. The correction consisted of detecting the values of the angles associated with the supine positions within these initial 10 min and subtracting these values to correct the sleep and stand angles of the remaining data. Both the initial sleep and stand angles of the corrected position were 90° after the correction for the supine sleep position.

### 2.4. Discretization of Sleep and Stand Angles

The discretization of the sleep and stand position monitoring angles was performed to compare the positions obtained from the smartphone with the video-validated positions from the PSG system. To classify the angles into the four sleep positions, threshold values of −140°, −40°, 60° and 120° were used to segment the 360° circle in the X-Z plane, as described in Figure 1b,c. Threshold values of −135°, −45°, 45° and 135° (Figure 1d) were used to segment the 360° circle in the Y-Z plane (Figure 1b) and classify the stand angles into two categories: standing (either stand or headstand) and lying. The thresholds used for the discretization of the X-Z and Y-Z planes were empirically set to mimic the criteria used by the sleep technicians to score the sleep position from the PSG video. Since a sleep position could not occur at the same time as a standing position, classification as standing overrides a simultaneously classified sleep position. For this reason, an extra rule was applied to determine the stand position to ensure that it was calculated with a non-close-to-zero vector module in the Y-Z plane. This rule was based on the following equation:

(3) $$\frac{\left|{\vec{a}}_{YZ}\right|}{\left|\vec{r}\right|}\geq Thld2\;\&\;\frac{\left|{\vec{a}}_{XZ}\right|}{\left|\vec{r}\right|}<Thld3$$

where Thld2 and Thld3 equal 0.8; ${\vec{a}}_{XZ}$ is the vector $\vec{a}$ described in step 2.1; ${\vec{a}}_{YZ}$ is the vector $\vec{a}$ described in step 2.2; and $\vec{r}$ is the vector $\vec{r}$ described in step 3.1, all of them in the subsection “Sleep Position Monitoring: Sleep and Stand Angles”.

### 2.5. Sleep Position Validation: Agreement by Patient

To investigate the agreement between the PSG and the smartphone, and the effect of the discretization (detailed in subsection “Discretization of Sleep and Stand Angles”), the smartphone initial correction (detailed in step 3.2 of the subsection “Sleep Position Monitoring: Sleep and Stand Angles” in this study) and the PSG video correction, we made three different comparisons for each patient:

1. The automatic smartphone accelerometry position vs. the validated hospital position: this analysis was made to compare the position obtained from the discretized smartphone angles, without the initial position correction, to the hospital video-validated reference position.
2. The automatic smartphone accelerometry corrected position vs. the validated hospital position: this analysis was made to compare the position obtained from the discretized smartphone angles with the initial position correction to the hospital video-validated reference position.
3. The automatic hospital position vs. the validated hospital position: this comparison served to understand how the corrections introduced by sleep technicians can improve the automatic sleep position from the PSG system.

For each of these pairwise comparisons, the percentage agreement between the two sleep positions was calculated for each patient. The sleep position vectors were compared sample-by-sample with the following equation:

(4) $$\%\mathrm{Agree}=100\cdot \frac{\sum_{i=1}^{N}{\;[\mathrm{Pos}}_{i}^{\mathrm{test}}=={\mathrm{Pos}}_{i}^{\mathrm{ref}}]}{N}$$

where ${\mathrm{Pos}}_{i}^{\mathrm{test}}$ is the vector with the position to be tested from the three comparisons; ${\mathrm{Pos}}_{i}^{\mathrm{ref}}$ is the reference position vector, which is the hospital video-validated position; and *N* is the total number of samples.

### 2.6. Sleep Position Validation: Agreement by Position

To validate how accurately each position was detected, we calculated the confusion matrix comparing sample-by-sample the four sleep positions and the stand position between the automatic smartphone accelerometry corrected position (test) and the validated hospital position (reference). The confusion matrix explains how many minutes were correctly and incorrectly classified for each position.

To assess how well each position was detected, we calculated the sensitivity (Se), specificity (Sp), positive predictive value (PPV), negative predictive value (NPV), and accuracy (Acc) from the information in the confusion matrix with the equations below:

(5) $$\mathrm{Se}=\frac{TP}{TP+FN}$$

(6) $$\mathrm{Sp}=\frac{TN}{TN+FP}$$

(7) $$\mathrm{PPV}=\frac{TP}{TP+FP}$$

(8) $$\mathrm{NPV}=\frac{TN}{TN+FN}$$

(9) $$\mathrm{Acc}=\frac{TP+TN}{TP+TN+FP+FN}$$

where TP denotes the minutes in which the position is correctly detected in both the smartphone and PSG devices (e.g., $Right_{PSG}\;\&\;Right_{Smartphone}$); TN the minutes in which the position is correctly not detected in both devices (e.g., $no\mathrm{t-R}ight_{PSG}\;\&\;\mathrm{not-R}ight_{Smartphone}$); FN the minutes in which the position is incorrectly detected as another position in the test device (e.g., $Right_{PSG}\;\&\;\mathrm{not-R}ight_{Smartphone}$); and FP the minutes in which the position is incorrectly detected as the specific position in question in the test device (e.g., $no\mathrm{t-R}ight_{PSG}\;\&\;Right_{Smartphone}$).

### 2.7. Sleep Position Characterization: Angle Distribution

To understand the interaction between the sleep and stand angles and the discretization limits described in the subsection “Discretization of Sleep and Stand Angles”, we used the discretized automatic smartphone corrected position to group each sample of the sleep angle into four categories (supine, prone, left, and right) and the samples of the stand angle, which belonged to the stand position into one category (stand).

For each of these five categories, we calculated the mean angle, the standard deviation (std), and the percentage of time spent by the patient around a window of ±5°, ±10°, ±15°, ±20°, and ±25° from the corresponding PSG reference angle. These PSG reference angles were set according to the axis direction of the smartphone accelerometry used in this study. The reference angles were 90° in the supine position, 0° in the left position, ±180° in the right position, and −90° in the prone position for the sleep angle (Figure 1c); and 0° and ±180° for the stand angle (Figure 1d). The equation used to calculate the percentage of angles recorded around these windows centered at the reference angle position is as follows:

(10) $$\%\mathrm{Angles}=100\cdot \;\frac{\sum_{i=1}^{N}\left[{\mathrm{Win}}^{low}\leq {\mathrm{Angle}}_{i}\leq {\mathrm{Win}}^{high}\right]}{N}$$

where ${\mathrm{Win}}^{low}$ and ${\mathrm{Win}}^{high}$ are the limits of the reference threshold ± the window value in degrees; *N* the number of angle samples; and ${\mathrm{Angle}}_{i}$ each of the samples with an angle value.

### 2.8. OSA Events Related to Sleep Position Angle

To address the relevance of the sleep position to the occurrence of OSA events, we used the OSA events from the simultaneous PSG study and assessed their distribution across the different sleep positions in the database.

For each patient, we calculated the percentage of time spent in a specific sleep position angle with the following formula:

(11) $${\%\mathrm{Position}}_{\theta }=100\cdot \;\frac{\sum_{i=1}^{N}{\mathrm{Position}}_{i}[{\mathrm{Angle}}_{\theta }^{low}\leq {\mathrm{Position}}_{i}\leq {\mathrm{Angle}}_{\theta }^{high}]}{N}$$

where ${\%\mathrm{Position}}_{\theta }$ provides the percentage of time spent in a specific sleep angle *θ*; *N* is the number of sleep position samples available; ${\mathrm{Position}}_{i}$ represents each sleep position sample matching the criteria between brackets; and the ${\mathrm{Angle}}_{\theta }^{low}$ and ${\mathrm{Angle}}_{\theta }^{high}$ are the thresholds used around each sleep angle *θ*, forming a window of 15° (*θ* ± 7.5°). The evaluated sleep angles *θ* ranged from −180° to 180° with 1° increase.

We also calculated the percentage of OSA events in a specific sleep position angle for each patient. To do this, we took each PSG event in the database and assigned to the event the median angle value from all sleep position angle samples which occurred during the event. We then determined the percentage of events occurring at a particular angle with the following formula:

(12) $${\%\mathrm{Event}}_{\theta }=100\cdot \;\frac{\sum_{i=1}^{N}{\mathrm{Event}}_{i}[{\mathrm{Angle}}_{\theta }^{low}\leq {\mathrm{Event}}_{i}\leq {\mathrm{Angle}}_{\theta }^{high}]}{N}$$

where ${\%\mathrm{Event}}_{\theta }$ is the percentage of events in a specific sleep angle *θ*; *N* is the number of events available; ${\mathrm{Event}}_{i}$ represents the median angle linked to an event matching the criteria between brackets; and the ${\mathrm{Angle}}_{\theta }^{low}$ and ${\mathrm{Angle}}_{\theta }^{high}$ are the thresholds used around each sleep angle *θ* with the same window and resolution as (11).

Finally, to determine the relationship between the occurrence of the events and the sleep position, two variables were calculated linked to their angle of occurrence: the local AHI and the ratio between the percentage of events and the percentage of position. These variables allowed us to estimate the severity of the OSA and to determine the sleep position angles with a greater incidence of events in comparison to the amount of time spent in that sleep position. The formulas used to calculate these two variables were as follows:

(13) $${\mathrm{Local}\;\mathrm{AHI}}_{\theta }=\frac{\sum_{i=1}^{N}{\mathrm{Event}}_{i}[{\mathrm{Angle}}_{\theta }^{low}\leq {\mathrm{Event}}_{i}\leq {\mathrm{Angle}}_{\theta }^{high}]}{\frac{1}{720,000}\cdot \sum_{j=1}^{M}{\mathrm{Position}}_{j}[{\mathrm{Angle}}_{\theta }^{low}\leq {\mathrm{Position}}_{j}\leq {\mathrm{Angle}}_{\theta }^{high}]}$$

(14) $${\mathrm{Ratio-\%Event}/\%\mathrm{Position}}_{\theta }=\frac{{\%\mathrm{Event}}_{\theta }}{{\%\mathrm{Position}}_{\theta }}$$

where the ${\mathrm{Local}\;\mathrm{AHI}}_{\theta }$ provides an estimation of the AHI in a specific sleep angle *θ*; *N* is the number of events available; ${\mathrm{Event}}_{i}$ represents the median angle linked to an event matching the criteria between brackets; ${\mathrm{Angle}}_{\theta }^{low}$ and ${\mathrm{Angle}}_{\theta }^{high}$ are the thresholds used around this angle with the same window and resolution as (11); the coefficient $\frac{1}{720,000}$ is used to normalize the position to hours, taking into account the sampling frequency of the smartphone (200 Hz) and the conversion from seconds to hours (1 h = 3600 s); M is the number of sleep position samples available; ${\mathrm{Position}}_{i}$ represents each sleep position sample matching the criteria between brackets; the ${\mathrm{Ratio-\%Event}/\%\mathrm{Position}}_{\theta }$ provides information about the occurrence of events at a specific sleep angle *θ*; and ${\%\mathrm{Event}}_{\theta }$ and ${\%\mathrm{Position}}_{\theta }$ are the results from Equations (11) and (12) for each specific angle *θ*.

To avoid high-value artifacts due to denominators being close to 0 in both the ${\mathrm{Local}\;\mathrm{AHI}}_{\theta }$ and ${\mathrm{Ratio-\%Event}/\%\mathrm{Position}}_{\theta }$ variables, a minimum denominator value is used in both equations, which is 10 min for the ${\mathrm{Local}\;\mathrm{AHI}}_{\theta }$ and 1% for the ${\mathrm{Ratio-\%Event}/\%\mathrm{Position}}_{\theta }$. Moreover, the ${\mathrm{Local}\;\mathrm{AHI}}_{\theta }$ is smoothed with the mean value calculated from the window containing the previous and following two values.

## 3. Results

### 3.1. Sleep Position: Angles and Discretization

**Figure 2:**
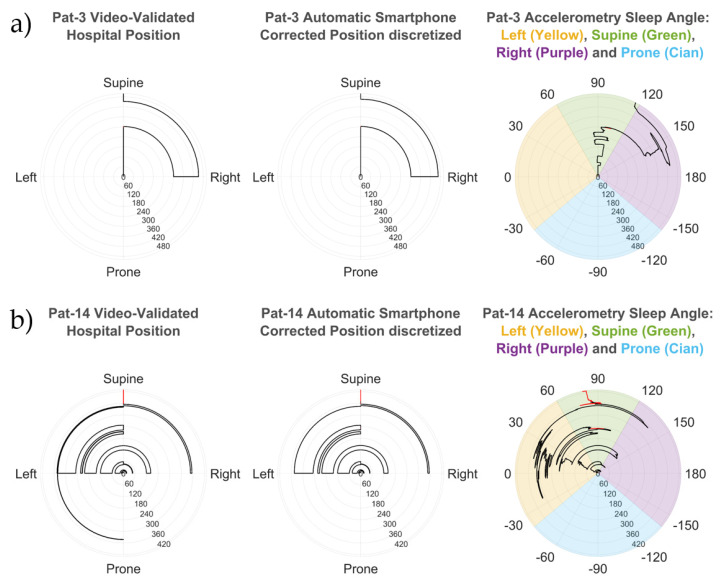
Patient 3 (**a**) and patient 14 (**b**) comparison between the hospital video-validated position (left polar plot in both patients) and the automatic smartphone corrected position (center polar plot in both patients) discretized from the accelerometry sleep angle (right polar plot in both patients). All polar plots indicate time in the radial axis (minutes) and position or angle value in the circumference. The stand position, if present, is displayed as a red line. The colored areas in the angle polar plots show the thresholds used to discretize the accelerometry sleep angle for the four different sleep positions (supine, prone, left, and right). The accelerometry angle correctly identifies all position shifts (even in difficult cases, such as patient 14), and provides patient sleep position with better resolution, which can reveal subtle angle variations not identifiable in the discretized positions (such as patient 3).

**Figure 3:**
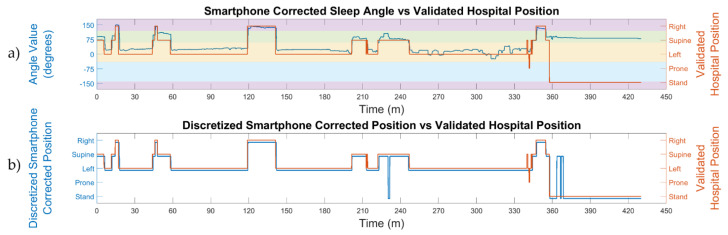
Comparison between the sleep position angle calculated from the smartphone accelerometry ((**a**), blue line) and the discrete video-validated hospital position ((**a**), orange line) for patient 14. The automatic smartphone discretized position ((**b**), blue line) is obtained from the smartphone accelerometry sleep angle for the four sleep positions and compared to the video-validated hospital position ((**b**), orange line). The smartphone stand position is calculated from the stand angle and shown together with the sleep positions. The color bands in the top image show the thresholds used to discretize the accelerometry sleep angle for the four different sleep positions (supine, prone, left, and right). The accelerometry angle correctly determines the movement of the patient and provides an accurate sleep position.

Two examples of the sleep angle calculated from the smartphone are shown in the polar plots for patients 3 and 14 in Figure 2 and overlaid onto the video-validated hospital position for patient 14 in Figure 3. The smartphone discretized angle from patient 3 (Figure 2a) shows perfect alignment with the manually video-validated position from the hospital PSG. Moreover, the sleep angle showed multiple subtle position changes which were not observable from the discrete validated hospital position data. Similar alignment is observed in patient 14 (Figure 2b), a complex case with multiple position shifts that were all properly detected.

Figure 3a plots the sleep angle and the hospital video-validated position, with close agreement observed between the two. However, the changes detected by the smartphone accelerometry are of greater resolution than those recorded as the video-validated position. The agreement between the automatic discretized smartphone corrected position and the hospital video-validated position can be seen in Figure 3b. Again, the smartphone detected some small stand and supine positions which were not defined in the validated hospital position. Nevertheless, most of the sleep positions were well identified by both devices.

### 3.2. Sleep Position Performance: Patient Overlap

Table 1 shows the amount of time, in minutes and as a percentage, that patients spent in each position for the discretized automatic smartphone corrected position and the manually video-validated hospital position. The prone position occurs infrequently, whereas the supine position is the most common (1.9% vs. 53.8% total prevalence). The same pattern is seen in the smartphone corrected position, with a prevalence of 0.8% and 55% for prone and supine positions, respectively. All patients slept for between 5 and 9 h, which is enough time to extract conclusions from the recorded data.

Table 2 shows the percentage of agreement between the three different comparisons made with the validated hospital position (reference) data. The automatic smartphone corrected position presents an average agreement of 95.9% with the reference data, ranging from 67.4% to 100%. Table 2 also shows the effect of the self-correction of the initial position, which increases the average agreement with the reference data by 1.5%. This is of relevance in patient 14, whose agreement varies from 81.6% to 96%. We can also see that the automatic hospital position agrees, on average, only 83.1% of the time with the video-validated hospital position, ranging from 52.5% to 100%, indicating that video validation was needed to properly detect the patients’ sleep position.

### 3.3. Sleep Position Performance: Position Overlap

To evaluate the detection of each position and the differences that appeared between them, a confusion matrix was built (Table 3) to compare the automatic smartphone corrected position and the video-validated hospital position. This confusion matrix shows the minutes spent at each position by all patients. Table 3 allows us to determine the interactions within the positions. For instance, it is possible to see that 8155.3 min (almost 136 h) of sleep were used to validate the performance of smartphones as sleep position monitors. It is of note that the diagonal of the table contains the larger values, representing 95.6% of the total minutes. This indicates that the correlation between the smartphone and the PSG is very high.

To determine the agreement between positions in greater detail, the Se, Sp, PPV, NPV, and Acc were calculated and are shown as percentages in Table 4. This table illustrates the high level of accuracy achieved by the smartphone in determining all sleep positions. The sensitivity values are very good in all cases except for the prone position, which was negatively affected by the low number of minutes spent in that position. The specificity values, the positive predictive values, and the negative predictive values for all the sleep positions are very good. On average, the overall system accuracy was 98.2%.

### 3.4. Sleep Position Characterization: Angle Distribution

**Figure 4:**
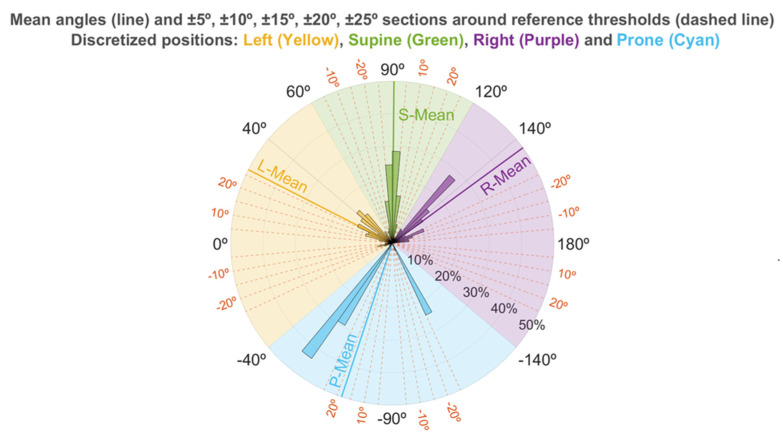
Percentage of minutes found at each sleep angle, classified in 5° segments, with respect to the total minutes for each position. Mean angles and ±5°, ±10°, ±15°, ±20°, ±25° windows around the reference angles are also shown.

Figure 4 illustrates how the angles calculated for the sleep position are distributed within a 360° circle. The 5° regions used show how the supine position has a centered distribution, whereas both the left and right, and prone, positions are more dispersed. This pattern is also apparent in Table 5, with the supine position being the only position having the mean value centered at its reference discrete angle. The mean value is 90.6°, while the reference supine value is 90°. The supine position also presents the lowest dispersion (smallest standard deviation). All other sleep positions have mean values that differ from the reference and higher standard deviations. This explains the wider range of different orientations observed for those positions. The stand position has a closer mean value (166.9°) to its reference value (180°), and its standard deviation is low enough for the positions to be not much dispersed.

The mean values and their high variability can also be seen in the percentage of angles found around the different windows for the reference angles each position should have. These values in Table 5 demonstrate how only 27.9% of the right positions, 35.4% of the left positions, and 2.6% of the prone positions were found in a ±25° window around their reference value, whereas 97.8% of the supine angles and 94.4% of the stand angles were found in the same window.

### 3.5. OSA Events Related to Sleep Position Angle

**Figure 5:**
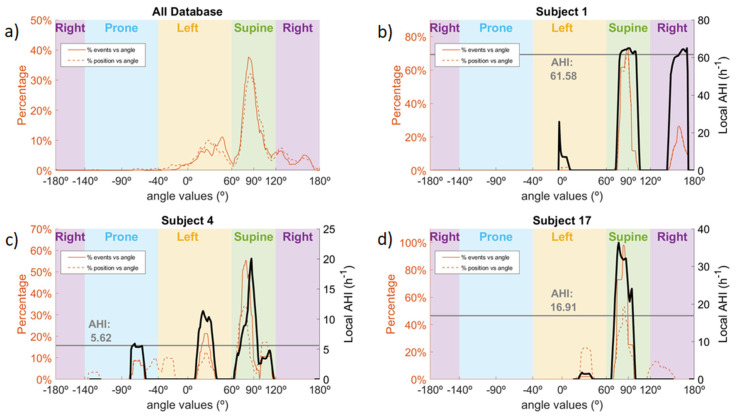
Percentage of OSA events and percentage of sleep position time in the database (**a**) with three individual examples (**b**–**d**). In (**a**), it is possible to observe the distribution of OSA events and sleep position time as a function of sleep angle for the whole database. The other three subfigures are examples of a clear non-pOSA patient (**b**), a clear pOSA patient (**d**) and an unclear pOSA patient (**c**), according to the local AHI displayed in black as a function of sleep angle. For each of the three subjects, the AHI of the whole night is shown with a grey line.

A total of 4047 events were recorded in the database. The lowest number of events, 47, occurred in subject 4, and the highest number of events, 633, occurred in subject 7. Of the 4047 events, 2439 (60%) occurred in the discrete supine position, 1039 (26%) in the discrete left position and 564 (14%) in the right position. Only five events occurred in the prone position, all in subject 4. Figure 5 shows the distribution of events in the whole database and the sleep position time distribution. In Figure 5a, it can be observed that most of the events occurred in the supine position, even though a lower percentage of the sleep time was spent in that position. Overall, most events and sleep time were spent in the discrete supine and left sleep positions. Of note is the fact that most events occurring in the left discrete position were very close to the threshold value used to differentiate between left and supine discrete positions, whereas the percentage of sleep time is more distributed within the left discrete position. In addition, three examples of different subjects from the database are shown (Figure 5b–d). Subject 1 is a clear case of non-pOSA, since the local AHI does not vary depending on the sleep position. In this case, we can see that the local AHI from the whole night is almost the same as the local AHI for both the supine and right sleep positions, and the local AHI for the left position could be neglected, since less than 2% of the time was spent at that position. Subject 17 is a clear case of pOSA, since the local AHI for the supine sleep position is much higher than the local AHI for the left sleep position. Finally, subject 4 is a non-clear case of pOSA, with differences between the supine position, with a local AHI of 20, and non-supine positions with a local AHI between 5 and 10.

**Figure 6:**
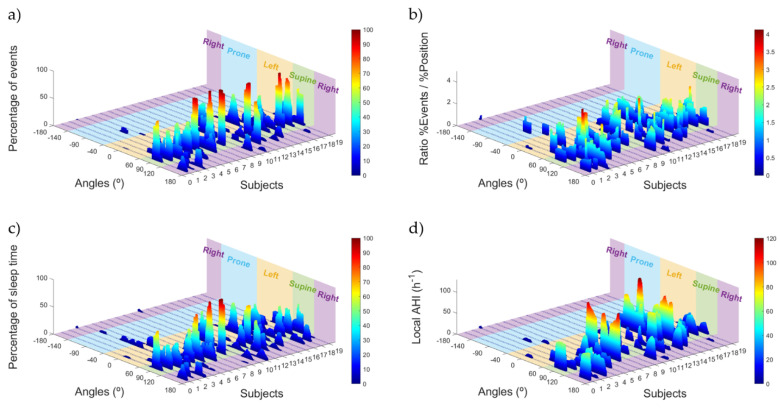
Percentage of OSA events (**a**), the percentage of sleep position time (**c**), the ratio between the percentage of OSA events and the percentage of sleep position time (**b**) and the local AHI (**d**) for each sleep position angle and for each of the subjects of the database. The four discrete sleep position areas have been colored and are shown in the 3D plot.

Figure 6 shows the distribution of OSA events and sleep position time as percentages, as well as the two variables used to detect pOSA and OSA severity: the ratio between the percentage of events and the percentage of sleep position time, and the local AHI, both as a function of specific sleep angle. In Figure 6a, showing the percentage of events, we can see that many subjects have most events in the supine position, with the examples of subjects 6, 8, 9, 13, 17, and 18 having almost 100% of events in this position. Conversely, there are subjects, such as 11 and 14, that have approximately 60% of events in the left position.

Figure 6c shows that there is a higher dispersion of sleep position time compared to the percentage of events. There are subjects, such as 8 and 9, who favor a supine sleep position, but more lateral sleep positions are also apparent. Figure 6d shows the local AHI, allowing us to distinguish the severity variability in the database. There are subjects with very severe OSA, such as 7, 14, and 15, and healthy subjects, such as 11. To complement this, Figure 6b shows the ratio between the percentage of events and the percentage of sleep position time spent as a function of angle. This variable allows us to determine the sleep position angles where the occurrence of events is higher than the percentage of time spent in that sleep position. For some subjects, this ratio is over 2, meaning that the number of events for that sleep position angle is, at least, doubled. Moreover, most of the high values of this ratio appear in the supine position, which agrees with the information in Figure 5a, where we can see more events in the supine position for the whole database than time spent in this position.

## 4. Discussion

### 4.1. Sleep Position: Discrete PSG vs. Discrete Sleep Angle

The aim of our study was to understand and quantify the reliability of retrieving sleep position using a smartphone and compare our results to those achieved with the gold-standard, video-validated PSG. We obtained a high average sleep position classification agreement of 95.9%, shown in Table 2. Although this agreement is true for most patients, two exceptions were patients 4 and 7, with agreement values of 67.4% and 89.4%, respectively. From Table 1, we can see that the difference in position designation for patient 4 was a result of an underestimation of the prone position in favor of the left position, and an underestimation of the supine position in favor of the right position. This mixed scoring happened because the angles associated with these left-prone misclassified positions and to the right-supine misclassified positions were very close to the position decision edge of the PSG. This resulted in the sleep technicians making a binary decision opposite to that of the smartphone. This situation suggests that more precise sleep position technology is desirable, since it would provide additional information to sleep technicians and would allow a more accurate detection of the sleep position. The same reasoning applies to the lower-than-average level of agreement observed in patient 7, whose case also belongs to the right-supine misclassification.

This binary-decision difference between the technicians and the smartphone system described for patients 4 and 7 also contributes to the lower sensitivity calculated for the right and prone positions observed in Table 3 and Table 4. Since the total number of minutes for the right position is higher, the impact of this misclassification is attenuated, but the impact on the prone position is very relevant, resulting in a Se value of 38.9%. Since the PSG promotes the supine sleep position [26,27] due to the multiple wires and equipment used for the test, it is likely that pure prone sleep positions do not occur. For this reason, the prone positions assessed in this study were close to the decision edge angle for the lateral positions. This situation produced a situation in which the sleep technicians made an opposite decision to the one that our smartphone system did. Yet, if more pure prone positions had occurred, the sensitivity of the prone position would have been increased and better numbers would have been achieved. This situation reveals the need to monitor sleep position with a higher resolution, as we propose in our study, in order to avoid possible misclassification and to provide the sleep technicians with more objective measures to assess sleep position.

In addition, the results shown in Table 2 and Table 4 confirm the excellent results seen in Table 1, where the average agreement value is high. The diagonal of the confusion matrix represents 95.6% of the total minutes assessed, indicating that the detection of the sleep position was reliable. This statement is reinforced by the values from Table 4, where the values of Se, Sp, PPV, NPV, and Acc are very high. Moreover, considering that the prevalence of the prone sleep position is very low in the population [54], the high accuracy found in this study is representative of the general population.

Finally, the small percentage and time differences observed in Table 2 and Table 3 for each patient and position can be explained by the sleep position transition differences between the automatic smartphone algorithm and the manual video-validated corrections on the PSG. Since the precision for manual correction is limited to the resolution which the proprietary software of the PSG system allowed, there exists an accumulated error at the exact moment of the position shift. This error accounts for there not being 100% agreement between each of the different comparisons in Table 2, and why some small time values between different positions appear in the confusion matrix (Table 3).

These results indicate that the position obtained from the smartphone accelerometry could be reliably used to monitor sleep position, since the agreement with the video-validated PSG was excellent.

### 4.2. Enhanced Sleep Position Monitoring

Although sleep position has been shown to be an important factor in sleep quality, few studies aim to evaluate the position distribution beyond the four-position classification used in the PSG [52,55].

The concept of enhanced sleep position monitoring refers to the increase of position resolution provided by the calculation of the sleep and stand angles. It is enhanced because the PSG only gives four discrete position values, and the angles allow us to assess all possible intermediate positions. In this study, we demonstrated how the discretized positions conceal small position shifts, such as those seen in Figure 2, which can be observed with the sleep angle.

The higher resolution of our smartphone data allowed us to illustrate the variability of the values in both lateral positions (Figure 4 and Table 5). Therefore, there could be right and left positions close to the supine position, where the risk of apneas occurring is increased in patients with pOSA. Additionally, most of the right and left positions detected in this study were not recorded within a ±25° window around the reference value for those positions. This pattern is easily seen in Figure 4, where the mean values for the left, right, and prone positions are outside the boundaries of the ±25° window. This indicates that the four-position classification system used in the PSG might not have a high enough resolution to properly characterize the different sleep positions, which is very important for determining the effect of position in sleep diseases such as OSA [52].

This idea is reinforced when analyzing the distribution of OSA events along the different sleep angles. In Figure 5a, we can observe that approximately 30% of events which happened in the database occurred in the discrete left sleep position, but most of them were very close to the threshold value between left and supine sleep positions. This indicates that the assessment of pOSA could be misleading when only four discrete sleep positions are used, since OSA events occurring at 60° and at 0° in the left position are classified as the same. The use of the sleep position angle would be a more robust and powerful measure, allowing the occurrence of events to be linked to a more accurate sleep orientation. The three images in Figure 5 corresponding to subjects 1, 4 and 17 are good examples of how powerful the sleep angle can be to assess pOSA. Images such as these would allow a clinician to directly compare the local AHI to the sleep position angle of occurrence. For instance, subject 1 is a clear case of a non-pOSA patient, since the local AHI does not vary depending on the sleep position, and the patient slept at least 30% of the time in a non-supine position (around 130 min from Table 1). Subject 17 is a clear case of a pOSA patient, since the variation of the local AHI is significant when comparing the supine position to the lateral positions. This subject would be classified as severe OSA (AHI > 30) if he slept only in supine position, whereas he would be classified as healthy (AHI < 5) if he slept only in the left or right positions. According to Table 1, this subject slept for approximately 140 min in non-supine positions, which is sufficient time to assess this differential behavior to determine pOSA. Finally, subject 4 is an example of a patient in whom the assessment of pOSA would be challenging. The subject exhibits different behavior in the supine position compared to the lateral and prone positions. Nevertheless, there are sleep orientations at approximately −40° in which no events occurred, and other orientations, such as −70° or 30°, in which many events occurred. Although the overall AHI for this patient was 5.62, we can see that the local AHI in the supine position would be approximately 15. In this case, the increased sleep position resolution provided by the sleep angle would allow a clinician to perform a better assessment of the severity of the patient.

In Figure 6, we complemented the local AHI information for each patient (Figure 6c) with the ratio between the percentage of events (Figure 6a) and percentage of sleep position time (Figure 6c). This ratio (Figure 6b) provides the information of the occurrence of events related to a specific sleep angle. This variable does not consider the severity of the illness, as the AHI does, but helps to better understand the increased or decreased occurrence of events related to a specific sleep angle. Ratio values below 1 indicate that fewer events occur compared to the time spent at that specific sleep position angle, whereas ratio values over 1 indicate the opposite. In Figure 6b it is possible to see that many subjects have ratio values over 2, indicating that the occurrence of events in this position is more than doubled. We can observe in subject 3, for example, that the ratio increases when going closer to the supine position. This behavior can only be seen with the increase of resolution provided by the sleep angle. The more detailed information allows us to observe that subject 3 would not have the same amount of events when sleeping in the right position with an angle of 180°, scoring a ratio value between 0 and 1, or close to 120°, which is the threshold between supine and right position, which scores a ratio value between 1.5 and 2. The increase of resolution proposed in this article, with the use of the sleep angle, would help clinicians to decide which treatment strategies would be most suitable for each patient, since it would allow more accurate determination of the severity of the OSA, as well as deeper insights into the effect of sleep position.

### 4.3. Smartphone Sleep Monitors: Portable mHealth Tools

In recent years, there have been multiple attempts to develop mHealth tools to monitor sleep-related breathing disorders. These attempts aim to reduce the number of sensors used in the PSG to provide similar information related to sleep quality. Some authors have proposed determining sleep quality by analyzing only the pulse oximetry [47,48]. These approaches obtained very good results, but lacked the ability to determine the breathing pattern, which is an important factor for differentiating between apneas and hypopneas [11]. To improve this situation, other studies tried to use only audio, or audio combined with a pulse oximeter to determine the breathing pattern [35,38,46]. In parallel, accelerometry has also played an important role in the mHealth field. Actigraphy has been used to monitor sleep stages and sleep quality [39,44,45], but other authors have used it to monitor respiration [41,56,57,58,59]. In our study, to determine the sleep position and calculate the sleep and stand angles we decided to use a smartphone, placed over the sternum with an elastic band, to acquire triaxial accelerometry.

The use of smartphones attached to the chest may discourage subjects from adopting a prone sleep position, due to their physical presence. Nevertheless, population studies show that the prone position has a small prevalence (7.3%) within the different sleep positions [54], so we would not expect the smartphone to have a large effect on the sleep position choice. Despite this, in previous studies with the same smartphone system we observed patients who slept a considerable amount of time in the prone position [41], and it is very likely that the reason for not sleeping in prone-like positions is the use of the PSG system [26,27], which has a lot of wires and promotes supine-like sleep positions. The size and weight of today’s smartphones are, at worst, similar to those of several pieces of commercial OSA monitoring equipment, and they are the same size and weight as the PSG equipment. As can be seen from Figure 1a, the smartphone used in our system, which measures 142 × 72.5 × 8.1 mm, is of similar size and characteristics to the PSG system used, and is smaller and lighter than multiple home sleep apnea monitors. We chose smartphones as they are available worldwide and contain many embedded sensors that are useful for this kind of analysis. Moreover, they already have multiple tools which make it possible to easily develop applications, which is very favorable for designing and implementing new technologies and algorithms. We are aware that there are some potential risks to using smartphones placed over the chest, including emissions and the heat which can be generated. For this reason, we used the smartphone in airplane mode and with Wi-Fi and Bluetooth deactivated, to reduce the emissions and heat generated, and to avoid any harm to the patient. None of the patients reported any issues related to these risks and reported a better comfort than when using the PSG. The elastic band used for this study allowed good sleep comfort and was tight enough to prevent any smartphone shift during the night, ensuring that the sleep position detection was not biased. Additionally, with the configuration used in this study, we consider that there would be no further risk beyond that entailed by the PSG system or any portable sleep apnea device.

In this study, we proposed an approach for using smartphones to reliably monitor sleep position. The results we obtained demonstrate that the performance of smartphones in detecting sleep position is, at a minimum, equivalent to the current gold-standard PSG. Moreover, we establish that smartphones are able to provide the sleep position with a higher resolution than the current PSG position evaluation.

Also of note is the fact that the algorithms proposed in this study incorporate the ability to self-calibrate the initial position. This is important because of the variation in patient anatomies.

In the future, smartphones could have the potential to precisely monitor sleep position while, at the same time, providing diagnosis for OSA [38,41,52]. Furthermore, they could even incorporate the ability to verify or apply positional therapy for several days, similar to what other devices already do [60,61]. Monitoring a patient’s sleep position for multiple days would allow the effect of any treatment to be assessed, and modified if necessary, in a timely manner.

## 5. Conclusions

Assessment of sleep position is an important step in polysomnographic (PSG) studies, since there is evidence that certain sleep positions or excessive position changes influence sleep quality. Nevertheless, sleep position is usually classified as one of only four different values: supine, prone, left, and right. In this study, we demonstrated how smartphones could be used as sleep monitoring devices. We calculated two different angles, which allowed us to determine the four sleep positions and the stand position with a higher resolution when compared to the PSG test. To validate the positions found with the smartphone, we performed simultaneous PSG-smartphone acquisitions in 19 patients. We then discretized the information from the smartphone angles into the four sleep positions to compare them with the manually video-validated positions from the PSG. We obtained a high average agreement (95.6%) in the detection of the positions and showed the dispersion of positions adopted by subjects for the non-supine sleep positions. In addition, we observed that the occurrence of OSA events in the lateral sleep positions varied depending on the distance of the sleep position from the supine position threshold. This highlights the fact that a higher resolution of sleep position is required to better assess pOSA.

The novel results presented in this study suggest that smartphones are promising mHealth tools for enhanced position monitoring at hospitals and at home.

## 6. Patents

The algorithms explained in this manuscript are under a process to recognize the industrial property.

## Figures and Tables

**Table 1:** Time spent in each position in minutes and as a percentage for both the video-validated PSG and the corrected smartphone positions, for each patient.

	Validated Hospital Position										Automatic Smartphone Accelerometry Corrected Position									
	Right		Supine		Left		Prone		Stand		Right		Supine		Left		Prone		Stand	
Patient	Min.	%	Min.	%	Min.	%	Min.	%	Min.	%	Min.	%	Min.	%	Min.	%	Min.	%	Min.	%
1	122.8	26.8	327.1	71.4	8.2	1.8	-	-	-	-	121.3	26.5	328.1	71.6	8.5	1.9	-	-	0.1	0.0
2	96.4	24.6	276.7	70.5	19.3	4.9	-	-	-	-	95.3	24.3	277.7	70.8	19.3	4.9	-	-	-	-
3	150.7	30.2	346.4	69.3	-	-	-	-	2.8	0.6	163.9	32.8	333.7	66.7	-	-	-	-	2.3	0.5
4	86.4	17.0	188.0	37.0	72.7	14.3	156.2	30.8	4.2	0.8	22.9	4.5	250.9	49.4	167.1	32.9	61.4	12.1	5.3	1.1
5	51.0	12.7	277.5	69.1	72.9	18.2	-	-	-	-	35.6	8.9	290.1	72.3	75.6	18.8	-	-	-	-
6	-	-	355.3	100.0	-	-	-	-	-	-	-	-	355.3	100.0	-	-	-	-	-	-
7	44.8	10.4	232.7	53.9	154.3	35.7	-	-	-	-	-	-	277.5	64.3	154.4	35.7	-	-	-	-
8	-	-	504.5	98.7	-	-	-	-	6.8	1.3	-	-	505.8	98.9	-	-	-	-	5.5	1.1
9	2.6	0.7	367.1	99.3	-	-	-	-	-	-	2.6	0.7	367.1	99.3	-	-	-	-	-	-
10	68.9	16.4	3.5	0.8	345.8	82.1	-	-	2.8	0.7	69.7	16.6	1.9	0.5	346.7	82.3	-	-	2.8	0.7
11	105.7	24.0	68.4	15.5	256.2	58.2	-	-	10.0	2.3	102.7	23.3	74.2	16.8	256.6	58.3	-	-	6.9	1.6
12	239.5	56.3	161.8	38.0	19.8	4.7	-	-	4.7	1.1	237.8	55.9	165.3	38.8	19.2	4.5	-	-	3.4	0.8
13	105.0	29.5	242.1	68.0	-	-	0.8	0.2	8.1	2.3	104.9	29.5	244.5	68.7	-	-	-	-	6.6	1.8
14	34.7	8.1	65.1	15.1	257.2	59.8	0.7	0.2	72.5	16.8	34.4	8.0	66.4	15.4	260.3	60.5	-	-	68.9	16.0
15	112.3	22.3	152.6	30.3	233.3	46.4	-	-	4.8	1.0	112.2	22.3	157.4	31.3	230.2	45.8	-	-	3.3	0.7
16	-	-	126.6	27.0	338.0	72.1	-	-	4.2	0.9	-	-	123.7	26.4	341.3	72.8	-	-	3.6	0.8
17	76.7	21.0	221.6	60.6	67.3	18.4	-	-	-	-	77.0	21.1	201.7	55.2	86.8	23.8	-	-	-	-
18	106.3	28.5	207.9	55.8	58.4	15.7	-	-	-	-	105.8	28.4	208.4	55.9	58.3	15.7	-	-	-	-
19	6.8	1.5	262.7	59.0	172.1	38.7	-	-	3.5	0.8	4.5	1.0	259.3	58.3	177.4	39.9	-	-	3.9	0.9
Total	1410.6	17.3	4387.4	53.8	2075.4	25.4	157.7	1.9	124.2	1.5	1290.5	15.8	4489.0	55.0	2201.8	27.0	61.4	0.8	112.6	1.4

**Table 2:** Percentage of position agreement between PSG and smartphone.

Patient	Automatic Smartphone Accelerometry Position vs. Validated Hospital Position	Automatic Smartphone Accelerometry Corrected Position vs. Validated Hospital Position	Automatic Hospital Position vs. Validated Hospital Position
1	99.4	99.5	94.5
2	99.7	99.7	91.6
3	90.0	96.8	89.6
4	61.1	67.4	58.0
5	95.0	95.3	75.6
6	100.0	100.0	100.0
7	89.4	89.4	58.8
8	99.4	99.8	98.7
9	100.0	100.0	100.0
10	99.2	99.2	75.1
11	96.5	96.5	86.1
12	98.4	98.6	95.1
13	98.2	99.2	97.5
14	81.6	96.0	68.8
15	99.1	98.4	52.5
16	94.7	95.8	78.4
17	93.4	93.3	97.6
18	99.4	99.4	99.4
19	99.0	98.0	61.2
Average	94.4	95.9	83.1

**Table 3:** Confusion matrix in minutes of the comparison between automatic smartphone corrected position and validated PSG position.

		Automatic Smartphone Accelerometry Corrected Position					Row Total	
	Position	Right	Supine	Left	Prone	Stand	Min.	%
Validated Hospital Position	Right	1268.8	139.6	1.6	0.0	0.6	1410.6	17.3
	Supine	19.9	4308.1	55.6	0.0	3.9	4387.4	53.8
	Left	1.9	23.4	2049.9	0.0	0.3	2075.4	25.4
	Prone	0.0	0.4	94.6	61.4	1.3	157.7	1.9
	Stand	0.0	17.6	0.1	0.0	106.5	124.2	1.5
Column Total	Min.	1290.5	4489.0	2201.8	61.4	112.6	8155.3	
	**%**	15.8	55.0	27.0	0.8	1.4		

**Table 4:** Sensitivity (Se), specificity (Sp), positive predictive value (PPV), negative predictive value (NPV) and accuracy (ACC) of the comparison between automatic smartphone corrected position and validated PSG position.

		Automatic Smartphone Accelerometry Corrected Position				
	Position	Se (%)	Sp (%)	PPV (%)	NPV (%)	Acc (%)
Validated Hospital Position	Right	89.9	99.7	98.3	97.9	98.0
	Supine	98.2	95.2	96.0	97.8	96.8
	Left	98.8	97.5	93.1	99.6	97.8
	Prone	38.9	100.0	100.0	98.8	98.8
	Stand	85.7	99.9	94.6	99.8	99.7
Average		82.3	98.5	96.4	98.8	98.2

**Table 5:** Sleep angle and stand angle characterization from the automatic smartphone corrected position. The reference angles are 90° for supine, ±180° for right, 0° for left and −90° for prone.

	Automatic Smartphone Accelerometry Classified by the Discretized Positions				
	Right	Supine	Left	Prone	Stand
Angle: Mean	143.9	90.6	27.0	−71.7	166.9
Angle: Standard deviation	14.0	8.7	21.2	26.6	12.1
% Angles: Reference angle ± 5° window	0.7	52.6	3.6	0	9.7
% Angles: Reference angle ± 10° window	5.9	80.2	7.0	0	24.8
% Angles: Reference angle ± 15° window	10.9	90.8	15.8	0	31.3
% Angles: Reference angle ± 20° window	17.4	95.9	27.1	0	76.8
% Angles: Reference angle ± 25° window	27.9	97.8	35.4	2.6	94.4

## Abbreviations

AASM	American academy of sleep medicine
Acc	Accuracy
AHI	Apnea hypopnea index
FN	False negative
FP	False positive
HRP	Home respiratory polygraphy
mHealth	Mobile health
Min	Minutes
NPV	Negative predictive value
OSA	Obstructive sleep apnea
Pos	Position
pOSA	Positional obstructive sleep apnea
PPV	Positive predictive value
PSG	Polysomnography
Se	Sensitivity
Sp	Specificity
Thld	Threshold
TN	True negative
TP	True positive
Win	Window
